# Research Progress of Ferroptosis in Cerebral Infarction

**DOI:** 10.1002/brb3.71192

**Published:** 2026-01-15

**Authors:** Yilan Fei, Qi Leng

**Affiliations:** ^1^ Nursing Department, Health Care Center, The First Affiliated Hospital Zhejiang University School of Medicine Hangzhou City China; ^2^ Health Care Center, The First Affiliated Hospital Zhejiang University School of Medicine Hangzhou City China

**Keywords:** cerebral infarction, ferroptosis, glutathione peroxidase 4 (GPX4), ischemic stroke

## Abstract

**Purpose**: To synthesize current mechanistic insights and translational progress on ferroptosis, a regulated, iron‐dependent, nonapoptotic cell death pathway in the pathophysiology and treatment of cerebral infarction (ischemic stroke), and to outline therapeutic opportunities and remaining gaps for clinical application.

**Method**: Narrative, focused review of preclinical and translational studies (in vitro, ex vivo, and in vivo ischemia/reperfusion and middle cerebral artery occlusion models), alongside emerging biomarker, nanocarrier, and gene/RNA‐based strategies reported up to 2025. Evidence was organized across five domains: (1) redox and lipid peroxidation biology; (2) iron metabolism and ferritinophagy; (3) mitochondrial dysfunction; (4) neuroinflammation and blood–brain barrier integrity; and (5) therapeutic development and early clinical exploration.

**Finding**: Ferroptosis in cerebral infarction is driven by glutathione depletion, glutathione peroxidase‐4 (GPX4) inactivation, and iron‐catalyzed lipid peroxidation of polyunsaturated phospholipids, with acyl‐CoA synthetase long‐chain family member‐4 (ACSL4) and lysophosphatidylcholine acyltransferase‐3 (LPCAT3) priming membranes for oxidative injury. Mitochondrial reactive oxygen species, iron–sulfur cluster instability, and cardiolipin oxidation amplify ferroptotic signaling, while ferroptosis–inflammation crosstalk (via damage‐associated molecular patterns and microglial activation) aggravates secondary injury and blood–brain barrier disruption. Candidate biomarkers (e.g., oxylipins, 8‐iso‐prostaglandin F2α, GPX4 fragments; gene pairs such as CDKN1A/JUN; NFE2L2 pathway readouts) show promise for patient stratification. Pharmacological approaches—including radical‐trapping antioxidants (ferrostatin‐1, liproxstatin‐1), iron chelation, and nuclear factor erythroid 2–related factor 2 (Nrf2) activation—consistently reduce infarct volume and improve function in animal models. Nanoparticle formulations enhance brain delivery of ferroptosis modulators, and RNA/gene‐targeted strategies (e.g., SLC7A11/GPX4/FSP1 axes; exosomal noncoding RNAs) expand the therapeutic toolkit. Clinically, iron‐modulating strategies in ischemic stroke suggest feasibility; however, dedicated, biomarker‐guided ferroptosis trials remain limited.

**Conclusion**: Ferroptosis represents a convergent, actionable mechanism of ischemic neuronal death and secondary brain injury. Multimodal interventions that combine lipid peroxidation control, iron homeostasis, mitochondrial protection, and inflammation resolution are biologically compelling. Key next steps include: validating real‐time biomarkers for patient selection and timing; optimizing brain‐penetrant delivery systems; integrating ferroptosis modulation with reperfusion therapies; and advancing rigorously designed phase II/III trials to establish efficacy and safety in defined stroke subtypes.

## Graphical abstract

Cerebral infarction triggers ischemia–reperfusion injury, leading to iron overload, oxidative stress, and mitochondrial dysfunction, which together initiate ferroptosis—an iron‐dependent, lipid peroxidation–driven form of neuronal death. Disruption of the System Xc^−^/GSH/GPX4 antioxidant axis and ACSL4‐mediated PUFA phospholipid enrichment accelerates membrane peroxidation. Ferroptotic neurons release DAMPs that activate microglia, amplify inflammation, disrupt the blood–brain barrier, and worsen secondary brain injury. Therapeutic strategies—including ferroptosis inhibitors, iron chelators, Nrf2 activators, nanocarrier‐based delivery systems, and RNA/gene‐targeted interventions—demonstrate strong neuroprotection in preclinical stroke models, offering promising translational potential for reducing infarct volume and improving functional outcome.

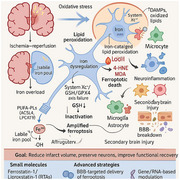

**Abbreviations**
ACSL4: acyl‐CoA synthetase long‐chain family member 4
BBB: blood–brain barrier
CoQ10: coenzyme Q10 (ubiquinone)
CSF: cerebrospinal fluid
DAMPs: damage‐associated molecular patterns
DFO: deferoxamine
DMT1: divalent metal transporter 1
ETC: electron transport chain
FSP1: ferroptosis suppressor protein 1 (also AIFM2)
GPX4: glutathione peroxidase 4
GSH: glutathione
HO‐1: heme oxygenase‐1
I/R: ischemia–reperfusion
IONPs: iron oxide nanoparticles
Lip‐1: liproxstatin‐1
LIPC (4‐HNE): 4‐hydroxynonenal (lipid peroxidation product)
LOX: lipoxygenase
MCAO: middle cerebral artery occlusion
MDA: malondialdehyde
MRI: magnetic resonance imaging
MRS: magnetic resonance spectroscopy
NCOA4: nuclear receptor coactivator 4 (mediator of ferritinophagy)
Nrf2 (NFE2L2): nuclear factor erythroid 2–related factor 2
PTGS2: prostaglandin‐endoperoxide synthase 2 (COX‐2)
PUFA: polyunsaturated fatty acid
ROS: reactive oxygen species
RTA: radical‐trapping antioxidant
SLC7A11: solute carrier family 7 member 11 (light chain of System Xc^−^)
System Xc^−^
: cystine/glutamate antiporter (SLC7A11/SLC3A2)
TfR1 (TFR): transferrin receptor 1
TFRC: transferrin receptor gene
tPA: tissue plasminogen activator (alteplase)

## 1 Introduction

Cerebral infarction, commonly referred to as ischemic stroke (IS), is a major cause of mortality and long‐term disability worldwide. It occurs when a cerebral artery is obstructed, leading to the deprivation of oxygen and nutrients in brain tissue, ultimately resulting in neuronal death and loss of neurological function. The pathological cascade following cerebral infarction involves a complex interplay of excitotoxicity, oxidative stress, inflammation, and various forms of cell death (Kuriakose and Xiao 2020). While traditional forms of cell death, such as necrosis and apoptosis, have long been studied in the context of cerebral infarction, recent research has brought attention to a novel form of regulated cell death, ferroptosis, as a key contributor to neuronal damage in IS (Salaudeen et al. 2024). Ferroptosis is an iron‐dependent, nonapoptotic form of cell death characterized by the accumulation of lipid peroxides and reactive oxygen species (ROS) (Chai et al. 2024). Unlike apoptosis or necrosis, ferroptosis is driven by iron‐catalyzed oxidative damage to cellular membranes, particularly phospholipids containing polyunsaturated fatty acids (PUFAs) (Zhang et al. 2022). Central to this process is the enzyme glutathione peroxidase 4 (GPX4), which normally protects cells by reducing lipid hydroperoxides to nontoxic lipid alcohols (Weaver and Skouta 2022; Manikandan et al. 2025). Inhibition of GPX4, depletion of glutathione (GSH), or disruption of cystine uptake through System Xc^−^ (Cystine/Glutamate Antiporter System) impairs the cell's antioxidant defenses, promoting uncontrolled lipid peroxidation and triggering ferroptosis (Li et al. 2022). The identification of ferroptosis as a critical mediator of neuronal injury in cerebral infarction represents a paradigm shift in stroke research, offering novel insights into pathophysiological mechanisms and opening new avenues for targeted therapeutic interventions (Jing et al. 2024).

Leads to the cerebral infarction, ischemia−reperfusion (I/R) injury primes a surge in oxidative stress and iron overload, creating a favorable environment for ferroptosis. Accumulating evidence from in vivo and in vitro studies has shown elevated markers of ferroptosis in ischemic brain regions, including increased iron accumulation, reduced GPX4 expression, and elevated levels of malondialdehyde (MDA) and 4‐hydroxynonenal (4‐HNE), hallmarks of lipid peroxidation (Lillo‐Moya et al. 2021). Furthermore, pharmacological inhibition of ferroptosis using agents like ferrostatin‐1, liproxstatin‐1, and iron chelators such as deferoxamine (DFO) has been shown to reduce infarct size, attenuate neuronal death, and improve functional recovery in experimental stroke models (Kailin et al. 2022). Recent advances in this field have also identified novel regulatory pathways and molecules involved in ferroptosis, such as the Nrf2 (nuclear factor erythroid 2–2‐related factor 2) antioxidant response, FSP1‐CoQ10 axis, and lipid metabolic enzymes like acyl‐CoA synthetase long‐chain family member 4 (ACSL4) and lysophosphatidylcholine acyltransferase 3 (LPCAT3), offering new insights into potential therapeutic targets (Tang et al. 2021; Yan et al. 2023). Moreover, the discovery of GPX4‐independent ferroptosis‐suppressing mechanisms expands the therapeutic landscape and underlines the complexity of redox regulation in the ischemic brain (Li et al. 2022). However, in this review, we will discuss the ferroptosis molecular mechanism and how it is involved in cerebral infarction. As well as ongoing investigations and progress into the molecular regulation and modulation of ferroptosis continue to advance our understanding of its role in ischemic brain injury, highlighting the need for translational strategies that can effectively harness this knowledge, thereby marking significant research progress in the field of ferroptosis in cerebral infarction.

## 2 Pathophysiology of Ferroptosis in Cerebral Infarction

**FIGURE 1:**
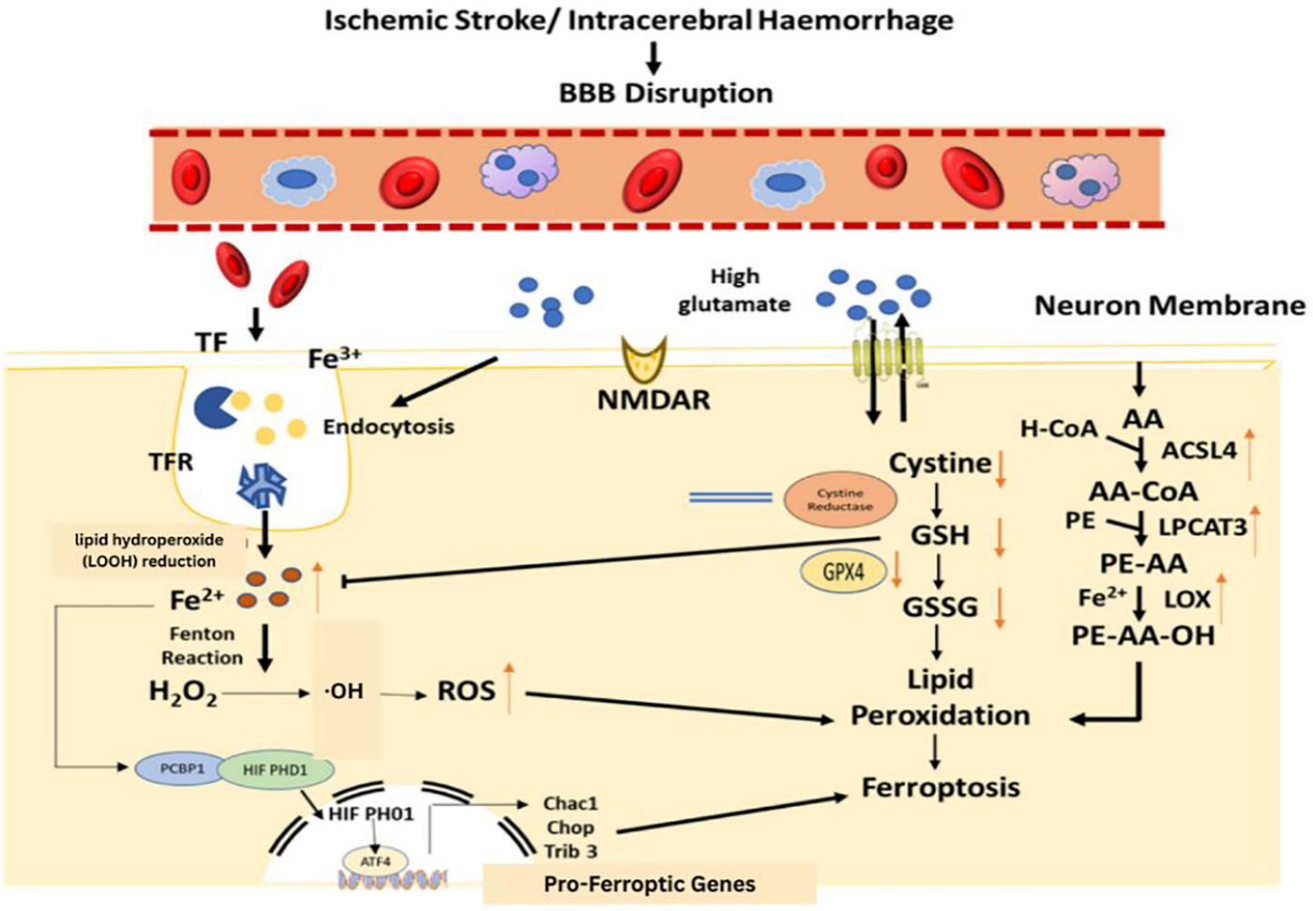
Mechanistic pathways underlying ferroptosis in cerebral infarction. This schematic illustrates the major molecular events that drive ferroptotic neuronal death following cerebral ischemia and reperfusion. Disruption of iron homeostasis increases intracellular Fe^2^^+^, which participates in the Fenton reaction to generate hydroxyl radicals (·OH) and amplify oxidative stress. In parallel, mitochondrial dysfunction during ischemia–reperfusion promotes excessive production of reactive oxygen species and destabilizes iron–sulfur clusters, further enriching the labile iron pool. Lipid peroxidation is initiated when ACSL4 and LPCAT3 incorporate polyunsaturated fatty acids (PUFAs) into membrane phospholipids, creating substrates for oxidation by lipoxygenases (LOX). Accumulating lipid hydroperoxides (LOOH) compromise membrane integrity and act as central executors of ferroptosis. Under physiological conditions, the System Xc^−^ transporter supplies cystine for glutathione (GSH) synthesis, enabling glutathione peroxidase‐4 (GPX4) to reduce LOOH to nontoxic lipid alcohols. During cerebral infarction, excitotoxic glutamate elevation and oxidative depletion of GSH inhibit the System Xc^−^/GSH/GPX4 axis, rendering neurons vulnerable to unrestricted lipid peroxidation. Ferroptotic cell death also interfaces with neuroinflammation: the release of damage‐associated molecular patterns (DAMPs) activates microglia, leading to cytokine production (e.g., IL‐1β, TNF‐α) that reinforces oxidative injury and ferroptosis. Together, these converging pathways iron dysregulation, mitochondrial ROS, impaired antioxidant defense, and inflammatory amplification drive ferroptosis as a key mechanism of neuronal injury in cerebral infarction.

Following the onset of cerebral ischemia and during reperfusion, the brain experiences a dramatic increase in oxidative stress, marked by the excessive generation of ROS. This oxidative burden overwhelms endogenous antioxidant defense systems and initiates lipid peroxidation, a key biochemical hallmark of ferroptosis (Salaudeen et al. 2024). At the same time, ischemic conditions disrupt iron homeostasis, leading to iron accumulation in neuronal tissues. In particular, excess ferrous iron (Fe^2^^+^) catalyzes the Fenton reaction, producing hydroxyl radicals (OH) that intensify oxidative damage by initiating the peroxidation of PUFA‐containing phospholipids in neuronal membranes (Fardin and Ali 2025). Central to ferroptosis regulation is GPX4, a lipid hydroperoxide detoxifying enzyme that uses GSH as a cofactor to convert toxic lipid peroxides into inert lipid alcohols (Long et al. 2023). During cerebral infarction, GPX4 activity is compromised due to intracellular GSH depletion and impaired cystine uptake. The latter is mediated by dysfunction of the System Xc^−^ transporter, which under normal physiological conditions imports cystine in exchange for glutamate. Under ischemic conditions, elevated extracellular glutamate concentrations caused by excitotoxicity inhibit System Xc^−^, further reducing cystine uptake, impairing GSH biosynthesis, and weakening the cell's antioxidant defense (Feng et al. 2023). Moreover, lipid metabolism enzymes such as ACSL4 and LPCAT3 play a significant role in incorporating PUFAs into phospholipids, making them prime substrates for peroxidation. The accumulation and oxidation of these lipid species compromise membrane integrity, resulting in iron‐dependent, nonapoptotic neuronal death, characteristic of ferroptosis (Sun et al. 2025). The mechanism and pathway of ferroptosis in cerebral infarction are shown in Figure 1.

Collectively, these converging disturbances in iron handling, redox balance, lipid metabolism, and antioxidant defense establish a cellular environment that strongly predisposes neurons to ferroptotic injury during cerebral infarction. The mechanistic framework outlined above underscores the centrality of ferroptosis within the broader I/R cascade. To substantiate these molecular pathways, a growing body of in vivo and in vitro research has provided direct experimental evidence demonstrating the occurrence and functional relevance of ferroptosis in stroke models.

### 2.1 Experimental Evidence Supporting Ferroptosis in Stroke Models

Building on this mechanistic foundation, experimental studies in established IS models—particularly the middle cerebral artery occlusion (MCAO) model—have validated the biochemical and morphological hallmarks of ferroptosis and clarified its contribution to neuronal loss after ischemic injury. Preclinical studies using animal models of cerebral ischemia, particularly the MCAO model, have provided compelling evidence for the role of ferroptosis in stroke pathology. These studies report increased levels of lipid peroxidation markers, such as MDA and 4‐HNE, along with suppressed GPX4 expression and elevated iron deposition in the ischemic brain (Tuo and Lei 2024). Crucially, pharmacological inhibition of ferroptosis using agents like ferrostatin‐1, liproxstatin‐1, and DFO (an iron chelator) has been shown to reduce infarct volume, preserve neuronal integrity, and improve post‐stroke neurological function (Youjun et al. 2025).

### 2.2 Ferroptosis and Secondary Injury Mechanisms

Beyond its role in initiating neuronal death, ferroptosis significantly contributes to secondary injury mechanisms. Ferroptotic neurons release damage‐associated molecular patterns (DAMPs), which activate microglia and astrocytes, leading to elevated production of proinflammatory cytokines (e.g., IL‐1β, TNF‐α) and the amplification of neuroinflammation (Kang et al. 2024). Additionally, ferroptosis exacerbates BBB disruption, facilitating the infiltration of peripheral immune cells and further promoting inflammatory damage (Xu et al. 2024). Mitochondrial dysfunction, another hallmark of I/R injury, adds to this cascade by generating excessive mitochondrial ROS and contributing to the disruption of iron–sulfur clusters, which accelerates ferroptosis progression (Xing‐Yu et al. 2024). Recent research has broadened our understanding of ferroptosis‐related mechanisms in cerebral infarction, including mitochondrial involvement, immune crosstalk, and translational biomarkers. These advancements are summarized in Table 1.

**TABLE 1:** Recent advances in understanding ferroptosis in cerebral infarction.

S. No.	Research area	Key insights and mechanisms	Significance	References
1	**Mitochondrial involvement in ferroptosis**	Mitochondria act as key amplifiers of ferroptosis under ischemic conditions. Ischemia−reperfusion leads to mitochondrial dysfunction, excessive ROS generation, and disruption of iron–sulfur clusters, which accelerates lipid peroxidation in neuronal cells.	Reveals a mitochondria‐centric pathway in ferroptosis initiation and suggests targeting mitochondrial redox balance.	Fu et al. (2023)
2	**Crosstalk between ferroptosis and inflammation**	Ferroptotic cell death releases DAMPs (damage‐associated molecular patterns), which stimulate microglial activation and inflammatory cytokine release (e.g., IL‐1β, TNF‐α), aggravating neuroinflammation and secondary neuronal injury post‐stroke.	Establishes ferroptosis as a contributor to both primary neuronal death and post‐ischemic inflammatory cascades.	He et al. (2024)
3	**Biomarker discovery for ferroptosis in stroke**	Novel CSF and plasma biomarkers such as oxylipins (oxidized lipid mediators), GPX4 fragments, and 8‐iso‐prostaglandin F2α have been identified as indicators of ferroptosis activity and oxidative lipid damage in stroke patients.	Paves the way for developing noninvasive, real‐time diagnostic tools for early detection and monitoring of ferroptosis.	Liu et al. (2025)
4	**Nanocarrier‐based ferroptosis inhibitor delivery**	Nanoparticle formulations (e.g., liproxstatin‐1‐loaded liposomes or polymeric carriers) have demonstrated enhanced BBB penetration, controlled drug release, and improved localization in infarcted brain regions.	Offers a promising strategy for targeted ferroptosis inhibition with improved pharmacokinetics and neuroprotection.	Shi et al. (2024)
5	**Clinical relevance and ongoing trials**	A first‐in‐human pilot trial (NCT05938447) is testing deferoxamine, an iron chelator, in patients with acute ischemic stroke and elevated serum iron. The goal is to evaluate safety, feasibility, and potential neuroprotective effects via ferroptosis modulation.	Marks early clinical translation of ferroptosis‐targeted therapy, bridging bench‐to‐bedside application.	Millán et al. (2021)

Complementing these advances, early phase clinical trials have begun exploring the safety and feasibility of ferroptosis modulators like DFO in acute IS patients with iron overload, marking a critical step toward clinical translation. Together, these findings underscore the expanding recognition of ferroptosis not only as a pathological hallmark but also as a modifiable and actionable target in cerebral infarction. Advances in mechanistic insight, biomarker discovery, targeted drug delivery, and therapeutic evaluation are collectively propelling this field toward innovative, ferroptosis‐centered approaches for stroke treatment and recovery. Collectively, these insights underscore the central role of ferroptosis in the pathophysiology of cerebral infarction. The convergence of oxidative stress, iron dysregulation, impaired redox homeostasis, and excitotoxicity drives ferroptotic neuronal death and secondary brain injury. Advances in mechanistic elucidation, biomarker discovery, drug delivery systems, and clinical testing have laid a robust foundation for translating ferroptosis‐targeted strategies into future therapeutic interventions (Liu et al. 2024). As the field continues to evolve, integrating ferroptosis modulation into stroke management may offer new hope for reducing morbidity and enhancing recovery in IS patients.

## 3 Mechanistic Integration, Research Progress, and Current Directions

The pathogenesis of ferroptosis in cerebral infarction is underpinned by several interconnected molecular mechanisms that reflect the complex biology of ischemic neuronal death. Emerging evidence has begun to elucidate how these mechanisms, namely, mitochondrial dysfunction, oxidative stress, neuroinflammation, iron metabolism, and impaired antioxidant defense, collectively orchestrate ferroptosis, offering novel avenues for targeted therapeutic intervention.

### 3.1 Mitochondrial Dysfunction as a Ferroptosis Amplifier

Mitochondria play a central role in ferroptosis by regulating redox balance, energy metabolism, and iron homeostasis. Under ischemic stress, mitochondrial respiration becomes impaired, leading to electron leakage at complexes I and III of the electron transport chain. This leakage produces excessive mitochondrial ROS, especially superoxide (O_2_^−^), which can be further converted into hydroxyl radicals via the Fenton reaction, exacerbated by iron overload (Tian et al. 2023). Moreover, iron–sulfur (Fe–S) cluster‐containing enzymes in the mitochondrial matrix, such as aconitase and complex I, undergo disintegration during oxidative stress, releasing redox‐active Fe^2^^+^ ions. This labile iron accelerates lipid peroxidation in mitochondrial membranes enriched in cardiolipin and other polyunsaturated phospholipids, contributing to ferroptotic signaling (Feng et al. 2024). Recent findings confirm that I/R disrupts mitochondrial morphology (e.g., outer membrane rupture, cristae loss), increases mitochondrial iron content, and reduces membrane potential hallmarks of ferroptosis. Current investigations are evaluating mitochondria‐targeted antioxidants like MitoQ, SkQ1, and SS‐31, along with iron chelators localized to mitochondria, to mitigate ferroptotic injury at its source (Feng et al. 2023; Salis Torres et al. 2024).

### 3.2 Neuroinflammation–Ferroptosis Crosstalk

Ferroptosis does not occur in isolation but intersects with the inflammatory landscape of the ischemic brain. Ferroptotic neurons release DAMPs, oxidized phospholipids, and lipid peroxidation products such as 4‐HNE and MDA. These molecules are sensed by pattern recognition receptors like TLR4 on microglia and astrocytes, triggering proinflammatory signaling cascades. Activated glial cells release cytokines such as IL‐1β, IL‐6, TNF‐α, and chemokines, contributing to secondary neuronal damage (Guo et al. 2025; Chen et al. 2024). Recent research contributes to potential therapeutic roles of various natural products in regulating neuroinflammation and ferroptosis. The results suggest that natural products have significant therapeutic potential in modulating the interaction between neuroinflammation and ferroptosis, making them potential treatments for Parkinson's disease (PD) (Guo et al. 2025). Future research should further validate the safety and efficacy of these natural compounds in clinical applications to develop novel therapeutic strategies for PD (Xu et al. 2024). Notably, this inflammatory microenvironment also feeds back into ferroptosis by increasing ROS production, inhibiting System Xc^−^, and reducing GPX4 expression. This bidirectional loop amplifies brain injury after stroke. Recent research demonstrated that blocking NF‐κB signaling attenuates both inflammation and ferroptotic neuronal death in ischemic models.  In addition, although studies have shown that ferroptosis incidence is highly correlated with LPO, only a small number of ferroptotic inhibitors target lipid‐rich mitochondria and cytomembrane with weak specificity. In the traditional sense, selenocysteine active sites in GPX4 and FSP‐1 play decisive roles in resisting ferroptosis. Therapeutically, dual‐pathway inhibitors targeting Nrf2 activation and NF‐κB suppression are under exploration to disrupt this pathogenic interface (Liu et al. 2024).

### 3.3 Biomarker Discovery: From Bench to Bedside

Recent research focuses on identifying biomarkers associated with ferroptosis to improve diagnosis, prognosis, and therapeutic targeting in cerebral infarction.

**Key Research Studies**

1. **Gene Pair Biomarkers in** IS
  - **Study Focus**: Identification of ferroptosis‐related gene pair biomarkers in IS and their roles in immune infiltration.
  - **Findings**: The gene pair CDKN1A/JUN was identified as a robust diagnostic biomarker, outperforming single‐gene markers. These genes are implicated in regulating ferroptosis during IS progression and are linked to immune cell infiltration, particularly plasma cells (Fan et al. 2022).
2. **Nfe2l2 as a Diagnostic Biomarker**
  - **Study Focus**: Investigation of ferroptosis‐related biomarkers in IS and their effects on immune infiltration using single‐cell RNA sequencing and animal models.
  - **Findings**: Nfe2l2 (nuclear factor erythroid‐derived 2‐like 2) was identified as a potential diagnostic biomarker. The study suggests that targeting the Nfe2l2/HO‐1 pathway may suppress ferroptosis and offer therapeutic benefits in IS (Fan et al. 2025).
3. **Oxidative Stress‐Ferroptosis Biomarkers**
  - **Study Focus**: Analysis of oxidative stress and ferroptosis‐related genes in stroke.
  - **Findings**: Four biomarkers, CDKN1A, GPX4, PRDX1, and PRDX6, were highlighted for diagnosing stroke and assessing treatment effects. These genes are involved in oxidative stress and ferroptosis pathways (Zhang et al. 2024).
4. **PTGS2 as a Diagnostic Marker**
  - **Study Focus**: Identification of diagnostic biomarkers at the intersection of oxidative stress and ferroptosis.
  - **Findings**: PTGS2 was found to be a differentially expressed gene of ferroptosis in IS. Inhibition of PTGS2 improved neurological outcomes and reduced infarct volume in animal models, suggesting its potential as a therapeutic target (Yuan et al. 2024).
5. **Mechanistic Reviews and Additional Biomarkers**

**Mechanisms**: Reviews have summarized the role of iron metabolism (e.g., serum ferritin, transferrin, hepcidin), glutamate metabolism, and antioxidant systems (e.g., GPX4, GSH, Nrf2 pathway) in ferroptosis during IS.
**Clinical Relevance**: Biomarkers such as serum ferritin, transferrin, hepcidin, and ceruloplasmin have been associated with stroke prognosis and ferroptosis activity in clinical and experimental settings (Deng et al. 2023).

### 3.4 Nanocarrier‐Based Therapeutic Delivery

- ROS‐Responsive Nanoparticles: Recent studies have developed ROS‐responsive nanoparticles that deliver ferroptosis inhibitors in a controlled manner. For example, a system combining human umbilical cord mesenchymal stem cells (Huc‐MSCs) and a ferroptosis inhibitor demonstrated synergistic neuroprotection in ischemic models by anchoring the therapeutic payload at the site of injury and releasing it in response to oxidative stress (Renshuai et al. 2024).
- Iron Oxide Nanoparticles (IONPs): IONPs have been explored for their ability to induce ferroptosis in cancer, but in the context of stroke, their application is more nuanced, acting as either imaging agents or, when combined with antioxidants/ferroptosis inhibitors, as neuroprotective carriers (Sant'Angelo et al. 2025).
- Functionalized Nanoparticles: Reviews highlight that functionalized nanoparticles (e.g., those decorated with targeting ligands) can cross the BBB and deliver ferroptosis modulators directly to ischemic brain tissue, enhancing efficacy and reducing systemic toxicity (Ziying et al. 2024).
- Natural Product‐Loaded Nanoparticles: Some natural antioxidants and ferroptosis inhibitors with poor bioavailability (such as N‐acetylcysteine and its derivatives) are being encapsulated in nanoparticles to improve brain delivery and therapeutic effect (Sun et al. 2023).

Nanoparticle‐based delivery systems, including ROS‐responsive, functionalized, and biomimetic nanoparticles, are at the forefront of preclinical research for targeting ferroptosis in cerebral infarction. These systems enable precise, BBB‐penetrant, and stimuli‐responsive delivery of ferroptosis inhibitors, iron chelators, and antioxidants, demonstrating significant neuroprotection in animal models. The field is rapidly evolving, with ongoing studies optimizing delivery vehicles and targeting strategies for future clinical translation (Sun et al. 2023).

### 3.5 Clinical Translation and Ongoing Trials

There are no registered or published clinical trials to date that specifically target ferroptosis in cerebral infarction (IS) in human patients (Dong et al. 2024; Maremonti et al. 2024). The field is still largely in the preclinical and translational research stage. Recent high‐impact studies, such as the 2025 investigation of the ATM inhibitor AZD1390, have demonstrated in vitro, ex vivo, and in vivo (animal) efficacy in reducing ferroptosis and protecting brain tissue after IS. However, these studies have not yet advanced to human clinical trials (Lee et al. 2025). Reviews from 2024 and 2025 emphasize the therapeutic potential of ferroptosis inhibitors (such as Fer‐1, DFO, and GPX4 activators) in stroke models, but also highlight that the lack of validated biomarkers for ferroptosis in humans and insufficient clinical data currently impede clinical trial development. Some iron chelators and antioxidants have been clinically tested for stroke, but not specifically as ferroptosis‐targeted therapies. As outlined in Table 2, ongoing efforts are focused on bridging this gap by refining biomarkers, improving delivery systems, and developing targeted interventions to accelerate the clinical translation of ferroptosis‐based therapies.

**TABLE 2:** Current research focus areas at a glance.

Focus area	What has been achieved	What current research is exploring	References
**Mitochondrial involvement**	Confirmed mitochondrial ROS and iron cluster disruption as triggers of ferroptosis	Development of mitochondrial‐targeted antioxidants to mitigate ferroptotic signaling	Feng et al. (2024)
**Inflammation‐ferroptosis crosstalk**	Demonstrated DAMP‐mediated cytokine release post‐ferroptosis	Investigating dual inhibitors targeting ferroptosis and inflammation (e.g., Nrf2‐NF‐κB axis modulators)	Yu et al. (2021)
**Biomarker discovery**	Identified oxylipins, GPX4 fragments, 8‐iso‐PGF2α as potential markers	Validation in human cohorts; integration into clinical diagnostic panels	van ’t Erve et al. (2015)
**Nanocarrier‐based drug delivery**	Liproxstatin‐1 nanoparticles improved BBB delivery and neuroprotection	Optimizing ligand‐targeted nanocarriers for precise ischemic tissue localization	Renshuai et al. (2024)

The expanding understanding of ferroptosis in IS has revealed a multidimensional network of mechanisms involving mitochondria, inflammatory signaling, redox imbalance, and iron metabolism. Recent progress in biomarker discovery, nanocarrier systems, and early phase clinical trials highlights the translational momentum of this field (Gowtham et al. 2024). Future research must now focus on integrating multitarget strategies, refining diagnostic tools, and accelerating the clinical application of ferroptosis‐targeted therapies to enhance stroke outcomes (Bu et al. 2021).

## 4 Therapeutic Strategies Targeting Ferroptosis in Cerebral Infarction

Recent advancements in understanding the molecular mechanisms of ferroptosis have catalyzed the development of targeted interventions aiming to mitigate ferroptosis‐induced neuronal death in IS. These strategies span from direct inhibition of lipid peroxidation to modulation of redox balance, iron metabolism, and gene expression (Chen et al. 2024). Below, we discuss the main therapeutic avenues currently under investigation.

### 4.1 Pharmacological Inhibitors of Ferroptosis

The most direct approach to mitigating ferroptosis is through small‐molecule inhibitors that interfere with its core molecular drivers: lipid peroxidation, iron accumulation, and GSH system failure.

#### 4.1.1 Lipid Peroxidation Inhibitors

Small molecules like ferrostatin‐1 (Fer‐1) and liproxstatin‐1 (Lip‐1) act as lipophilic radical‐trapping antioxidants, scavenging lipid peroxyl radicals (LOO•) and halting the peroxidation chain reaction. These agents preserve membrane integrity and mitochondrial function in rodent models of stroke, especially in MCAO and global ischemia models (Huang et al. 2023).

#### 4.1.2 GPX4 Modulators

GPX4 is a central enzyme that reduces lipid hydroperoxides (LOOH) to nontoxic lipid alcohols using GSH. Agents that upregulate GPX4, such as baicalein and selenium supplementation, enhance cellular defense mechanisms against ferroptosis. Moreover, targeting upstream molecules like SLC7A11, which imports cystine for GSH synthesis, offers indirect protection (Hu et al. 2019).

#### 4.1.3 Iron Chelators

Iron chelators such as:

- DFO: An FDA‐approved agent for iron overload, now in stroke trials.
- Deferiprone and deferasirox: Oral iron chelators with potential Central Nervous System (CNS) applications.
- VK‐28 and HBED: Brain‐penetrant experimental chelators (Mobarra et al. 2016; Entezari et al. 2022).

These chelators attenuate iron‐mediated ROS generation and disrupt the Fenton reaction (Fe^2^^+^ + H_2_O_2_ → Fe^3^^+^ + OH + OH^−^), a core contributor to ferroptosis initiation (Entezari et al. 2022).

#### 4.1.4 Nrf2 Pathway Activators

The Nrf2 pathway orchestrates the cellular antioxidant response. Activators like sulforaphane, dimethyl fumarate, and bardoxolone methyl promote the transcription of antioxidant genes, including SLC7A11, GPX4, HO‐1, and NQO1, restoring redox balance and ferroptosis resistance (Chen and Maltagliati 2018).

### 4.2 Combination Therapies: A Multitargeted Approach

Cerebral infarction is characterized by a complex interplay of oxidative stress, excitotoxicity, inflammation, mitochondrial dysfunction, and multiple forms of regulated cell death, including ferroptosis. Because these mechanisms are temporally and spatially interconnected, single‐target interventions often provide only partial neuroprotection. Recent preclinical studies increasingly support the use of combination therapies designed to modulate several pathogenic pathways simultaneously (Entezari et al. 2022; Chen and Maltagliati 2018). Such multimodal strategies are particularly relevant in I/R injury, where the abrupt restoration of blood flow triggers a surge in ROS, iron mobilization, lipid peroxidation, and inflammatory signaling conditions that strongly potentiate ferroptosis. Combination regimens pairing ferroptosis inhibitors with thrombolytics, anti‐inflammatory agents, iron chelators, or antioxidant compounds have shown synergistic benefits in experimental stroke models. For example, coadministration of radical‐trapping antioxidants with recombinant tissue plasminogen activator reduces I/R‐induced oxidative and lipid peroxidation damage without compromising thrombolytic efficacy (Huang et al. 2023; Mobarra et al. 2016). Similarly, iron chelation combined with microglial‐targeted agents attenuates both ferroptotic injury and inflammatory cascades. Other combinations, such as Nrf2 activators with ROS scavengers, enhance endogenous antioxidant defenses, whereas ferroptosis inhibitors paired with cytokine blockers mitigate secondary neuroinflammatory injury. Collectively, these emerging strategies highlight the therapeutic promise of coordinated, multitarget modulation of ferroptosis, inflammation, iron metabolism, and oxidative injury. Translating such regimens to clinical practice will require optimization of dosing windows, pharmacokinetics, and interactions with standard‐of‐care reperfusion therapies (Entezari et al. 2022; Briones‐Valdivieso et al. 2024). A summary of the representative combination has been shown in Table 3.

**TABLE 3:** Representative combination therapies targeting ferroptosis and complementary pathways in cerebral infarction.

Combination regimen	Mechanistic synergy	Experimental evidence/model outcomes	Key primary references
**Ferrostatin‐1 (Fer‐1) + alteplase (tPA)**	Fer‐1 scavenges lipid peroxyl radicals and prevents ferroptotic membrane damage; tPA restores cerebral perfusion. Combined use reduces I/R‐induced oxidative burst.	In MCAO models, Fer‐1 coadministration reduces infarct size and neuronal lipid peroxidation during post‐tPA reperfusion without impairing thrombolysis.	Entezari et al. 2022; Chen and Maltagliati 2018
**Deferoxamine (DFO) + minocycline**	DFO chelates Fe^2^^+^ and suppresses Fenton chemistry; minocycline inhibits microglial activation and downstream inflammatory cytokines.	Combined therapy reduces iron accumulation, microglial polarization, and neuronal ferroptosis in I/R models.	Briones‐Valdivieso et al. (2024)
**Sulforaphane + edaravone**	Sulforaphane activates Nrf2, enhancing GPX4, HO‐1, and GSH synthesis; edaravone scavenges ROS and inhibits lipid peroxidation.	Dual treatment enhances antioxidant capacity, reduces mitochondrial ROS, and improves neurological recovery.	Hu et al. 2019; Mobarra et al. 2016
**Liproxstatin‐1 (Lip‐1) + anti‐TNF agents**	Lip‐1 inhibits lipid peroxidation; TNF‐α blockade reduces neuroinflammation and suppresses cytokine‐driven ferroptosis amplification.	Demonstrated reduction of BBB disruption, cytokine burden, and ferroptotic neuron loss in rodent MCAO models.	Huang et al. 2023; Briones‐Valdivieso et al. 2024

### 4.3 Natural Compounds and Nutraceuticals

Natural compounds have demonstrated significant potential in modulating ferroptosis‐related pathways, offering promising therapeutic strategies for conditions like cerebral infarction. One of the key mechanisms involves activation of the Nrf2/ARE pathway, where several flavonoids such as quercetin, baicalin, and kaempferol enhance antioxidant defenses by upregulating genes like GPX4, a crucial enzyme that suppresses lipid peroxidation (Li et al. 2025; Shi et al. 2024). Additionally, iron chelation has emerged as a vital approach, with compounds like salvianolic acid A and the clinically used DFO effectively reducing iron overload, a central trigger of ferroptosis (Shi et al. 2024). Natural antioxidants such as baicalein and epigallocatechin gallate also play a pivotal role by directly scavenging ROS and inhibiting the chain reaction of lipid peroxidation, thereby preserving neuronal membrane integrity (Bi‐Tiao et al. 2025). Traditional Chinese Medicine and herbal formulations further contribute to ferroptosis regulation. For example, Danhong injection, comprising *Salvia miltiorrhiza* and saffron, has shown efficacy in improving outcomes in IS by modulating ferroptotic processes (Chen et al. 2024). Moreover, nonpharmacological interventions like moxibustion have demonstrated neuroprotective effects through the inhibition of ROS accumulation and ferroptotic neuronal death. Collectively, these natural approaches represent a multifaceted strategy for targeting ferroptosis in neurological disorders. A summary of these compounds and their specific mechanisms of action is provided in Table 4, offering a concise overview of their therapeutic relevance.

**TABLE 4:** Key natural compounds in ferroptosis inhibition for cerebral infarction.

S. No.	Compound	Source	Mechanism of action	Evidence in cerebral infarction	References
1.	Quercetin	Flavonoid	Antioxidant, inhibits lipid peroxidation, modulates Nrf2/GPX4	Shown to reduce ferroptosis and protect neurons in stroke models	Zheng et al. (2025)
2.	Baicalin/baicalein	Flavonoid (Scutellaria baicalensis)	Activates Nrf2/ARE, upregulates GPX4, reduces iron overload	Demonstrated neuroprotection in ischemic stroke	Li et al. (2025)
3.	Epigallocatechin‐3‐gallate (EGCG)	Green tea polyphenol	Antioxidant, inhibits lipid peroxidation	Shown to inhibit ferroptosis in CNS models	Zheng et al. (2025)
4.	Berberine	Alkaloid (Coptis chinensis)	Modulates iron homeostasis, antioxidant	Shown to protect against ischemic injury	Bi‐Tiao et al. (2025)
5.	Salvianolic acid A	Polyphenol (Salvia miltiorrhiza)	Iron chelator, inhibits lipid peroxidation	Reduces neuronal ferroptosis in hemorrhagic stroke	Shi et al. (2024)
6.	Icariside II	Flavonoid (Epimedium)	Activates Nrf2/GPX4, reduces oxidative stress	Shown to inhibit ferroptosis after ischemia	Zhang et al. (2024)
7.	Dl‐3‐n‐butylphthalide	Synthetic from celery seed	Antioxidant, modulates iron metabolism	Effective in animal stroke models	Xu et al. (2023)
8.	Ginkgolide B	Terpene lactone (Ginkgo biloba)	Antioxidant, anti‐inflammatory	Demonstrated efficacy in stroke models	Xu et al. (2025)
9.	Loureirin C	Flavonoid (Dragon's blood)	Antioxidant, modulates iron metabolism	Potential neuroprotective effects	Xu et al. (2023)
10.	Astragaloside IV	Saponin (Astragalus membranaceus)	Inhibits ferroptosis‐associated pathways	Shown to alleviate brain injury in stroke	Zhang et al. (2023)
11.	Resveratrol	Polyphenol (grapes, berries)	Inhibits ferroptosis‐induced cell death	Shown to reduce ischemia/reperfusion injury	Li et al. (2022)

These compounds are being tested in various animal models and may serve as long‐term maintenance therapy for high‐risk patients or stroke survivors to prevent secondary injury. These agents act through antioxidant, iron‐chelating, and antilipid peroxidation mechanisms, often modulating key signaling pathways such as Nrf2/GPX4. While preclinical data are encouraging, further clinical research is needed to translate these findings into effective treatments for stroke patients.

### 4.4 Gene and RNA‐Based Therapeutic Strategies

Targeting ferroptosis at the genetic and transcriptional level provides a more specific and durable strategy to modulate neuronal vulnerability.

#### 4.4.1 RNA‐Based Therapeutic Approaches

RNA‐based therapeutic strategies are gaining momentum as innovative approaches to modulate ferroptosis, particularly in the context of neurodegenerative and ischemic conditions such as stroke (Henninger and Mayasi 2019). Noncoding RNAs (ncRNAs), including microRNAs (miRNAs), long noncoding RNAs (lncRNAs), and circular RNAs (circRNAs), have emerged as key regulators of ferroptosis through their control over gene expression and signaling pathways (Wu et al. 2025; Zeyu et al. 2022). Among miRNAs, miR‐194 enhances cell survival and suppresses ferroptosis by targeting the repressor Bach1, thereby activating the Nrf2/HO‐1 antioxidant pathway. Similarly, miR‐210‐3p, when delivered via hypoxia‐conditioned exosomes, downregulates transferrin receptor expression, effectively limiting iron uptake and reducing ferroptosis. Another miRNA, miR‐23a‐3p, carried by mesenchymal stem cell (MSC)‐derived exosomes, mitigates ferroptotic damage by lowering oxidative stress and ferroptosis biomarkers (Wei et al. 2022). In terms of lncRNAs, Mir9‐3hg identified in bone marrow MSC‐derived exosomes suppresses ferroptosis by modulating RNA‐binding proteins and antioxidant enzyme expression. circRNAs, though still under active investigation, have shown dual roles, either promoting or inhibiting ferroptosis, suggesting their potential both as diagnostic biomarkers and therapeutic targets. Notably, the exosomal delivery of these ncRNAs from stem cells has shown substantial promise in preclinical models by attenuating ferroptosis and ameliorating ischemic injury. These findings underscore the therapeutic relevance of ncRNAs and pave the way for RNA‐based interventions in ferroptosis‐related neurological disorders (Zhang and Xie 2024).

#### 4.4.2 CRISPR‐Cas9 Gene Editing

Permanent disruption or repair of ferroptosis‐related genes (e.g., GPX4, SLC7A11, FSP1) using CRISPR could offer long‐term neuroprotection, especially in genetically predisposed individuals. This requires caution due to off‐target risks but holds immense promise. One landmark study used a CRISPR–Cas9 screening strategy to identify FSP1 (ferroptosis suppressor protein 1) as a potent inhibitor of ferroptosis. FSP1 was validated as a key target in both hemorrhagic and IS models, suggesting that gene editing of FSP1 or related pathways could modulate ferroptosis and influence stroke outcomes (Xu et al. 2023).

#### 4.4.3 Antisense Oligonucleotides

Antisense oligonucleotides (ASOs) can downregulate ferroptosis drivers such as p53, which suppresses SLC7A11, and NCOA4, which promotes ferritinophagy (iron release from ferritin). Early preclinical studies show positive outcomes (Liu and Gu 2022). A relevant study discusses the potential of ASOs to downregulate key ferroptosis drivers such as p53 and NCOA4, which are involved in promoting ferroptosis through suppression of SLC7A11 and ferritinophagy (iron release), respectively. Early preclinical evidence suggests that targeting these ferroptosis regulators with ASOs can mitigate neuronal death and cerebral ischemic injury by reducing iron overload and lipid peroxidation in stroke models (Xu et al. 2023).

### 4.5 Clinical Translation: Progress and Roadblocks

While preclinical data are encouraging, clinical translation remains in early phases. The first clinical trial (NCT05938447) is testing DFO in patients with acute IS and elevated iron markers. Initial goals are to evaluate safety, pharmacodynamics, and preliminary efficacy.

**Translational Challenges Include**:

- **Therapeutic window**: Ferroptosis inhibitors must be administered within hours of stroke onset.
- **Patient variability**: Stroke subtypes (lacunar, embolic, hemorrhagic) may respond differently.
- **Lack of real‐time biomarkers**: Hinders the identification of ferroptosis‐active patients.
- **Systemic side effects**: Especially with iron chelators or GPX4 modulators.

**Future Directions**:

- **Personalized medicine**: Tailoring treatments based on iron load, oxidative stress markers, and genomics.
- **Advanced neuroimaging**: Use of iron‐sensitive MRI, diffusion tensor imaging, and MRS to guide therapy.
- **Larger phase II/III trials**: Needed to validate efficacy and define clinical endpoints.

Therapeutic targeting of ferroptosis in cerebral infarction is advancing rapidly, integrating insights from redox biology, iron metabolism, and molecular pharmacology. The combination of traditional small‐molecule drugs, natural antioxidants, gene‐editing technologies, and nanocarrier systems offers a multidimensional arsenal against ischemic brain injury. As research transitions from bench to bedside, strategic efforts must prioritize clinical validation, patient stratification, and timely intervention to harness the full therapeutic potential of ferroptosis modulation.

## 5 Conclusion

Ferroptosis has emerged as a critical and distinct form of regulated cell death in the pathophysiology of cerebral infarction, offering a paradigm shift in our understanding of ischemic neuronal injury. Unlike necrosis or apoptosis, ferroptosis is characterized by iron‐dependent lipid peroxidation, compromised antioxidant defenses, particularly GPX4 inactivation and GSH depletion, and mitochondrial oxidative stress. These molecular hallmarks of ferroptosis are increasingly recognized as central contributors not only to primary neuronal death but also to secondary injury mechanisms, including blood–brain barrier disruption, neuroinflammation, and mitochondrial dysfunction in the ischemic brain.

Over the past decade, substantial progress has been made in dissecting the molecular architecture of ferroptosis in cerebral ischemia. Studies have delineated the roles of key players such as System Xc^−^, ACSL4, Nrf2, and iron‐regulatory proteins like ferritin, TfR1, and DMT1 in driving ferroptotic cascades. Moreover, mitochondrial ROS generation, iron–sulfur cluster instability, and ferritinophagy have been identified as amplifiers of ferroptotic injury in neurons and vascular endothelial cells. Importantly, these findings underscore that ferroptosis is not an isolated process but rather a converging node where redox imbalance, iron overload, excitotoxicity, and inflammation intersect.

From a translational standpoint, preclinical evidence is robust and promising. Pharmacological inhibition of ferroptosis through lipid ROS scavengers (ferrostatin‐1, liproxstatin‐1), iron chelators (DFO), GPX4 stabilizers, and Nrf2 activators has demonstrated consistent neuroprotective effects in rodent models of MCAO. These interventions have shown benefits such as reduced infarct volume, preserved neuronal integrity, improved behavioral outcomes, and delayed neurodegeneration.

Recent innovations such as nanocarrier‐based delivery systems, gene and RNA‐based therapies, and biomarker discovery are accelerating the transition of ferroptosis research from bench to bedside. The initiation of early phase human trials evaluating DFO in acute IS (NCT05938447) signals an encouraging move toward clinical translation. However, challenges remain in terms of drug delivery across the blood–brain barrier, defining optimal therapeutic windows, and stratifying patients based on ferroptosis susceptibility using biomarkers or imaging tools.

Looking ahead, future research should focus on:

- Clarifying the temporal dynamics of ferroptosis during the acute, subacute, and chronic phases of stroke.
- Integrating ferroptosis modulation with standard stroke therapies such as thrombolytics and neurorehabilitation.
- Personalizing interventions based on age, sex, iron status, and genetic background.
- Exploring ferroptosis‐inflammation crosstalk as a dual therapeutic target.
- Developing real‐time diagnostic tools to monitor ferroptosis in vivo.

In conclusion, ferroptosis represents not only a novel and actionable mechanism of neuronal death in cerebral infarction but also a promising target for therapeutic innovation. The research progress made thus far has laid a strong foundation, but continued interdisciplinary efforts spanning molecular neuroscience, pharmacology, biomaterials, and clinical medicine are essential to fully harness the therapeutic potential of ferroptosis‐targeted strategies in the fight against stroke‐induced brain injury.

## Author Contributions

Yilan Fei and Qi Leng: Participated in collecting, assessing, and interpreting the data. Made significant contributions to data interpretation and manuscript preparation. Yilan Fei and Qi Leng: Provided substantial intellectual input during the drafting and revision of the manuscript. All authors have read and approved the final manuscript.

## Funding

The authors have nothing to report.

## Conflicts of Interest

The authors declare that they have no financial conflicts of interest.

## Consent to Publish

The manuscript has neither been previously published nor is under consideration by any other journal. The authors have all approved the content of the paper.

## Data Availability

The data that support the findings of this study are available from the corresponding author upon reasonable request.
